# CD44 enhances invasion of basal-like breast cancer cells by upregulating serine protease and collagen-degrading enzymatic expression and activity

**DOI:** 10.1186/bcr3199

**Published:** 2012-05-23

**Authors:** Nicola Montgomery, Ashleigh Hill, Suzanne McFarlane, Jessica Neisen, Anthony O'Grady, Susie Conlon, Karin Jirstrom, Elaine W Kay, David JJ Waugh

**Affiliations:** 1 Centre for Cancer Research and Cell Biology, Queens University Belfast, 97 Lisburn Road, Belfast, BT9 7BL, Northern Ireland; 2 Department of Histopathology, Royal College of Surgeons in Ireland, Beaumont Hospital, Beaumont Road, Dublin 9, Ireland; 3 Division of Pathology, Department of Laboratory Medicine, Lund University, Malmo University Hospital, S-205 02 Malmo, Sweden

## Abstract

**Introduction:**

Basal-like breast cancers (BL-BCa) have the worst prognosis of all subgroups of this disease. Hyaluronan (HA) and the HA receptor CD44 have a long-standing association with cell invasion and metastasis of breast cancer. The purpose of this study was to establish the relation of CD44 to BL-BCa and to characterize how HA/CD44 signaling promotes a protease-dependent invasion of breast cancer (BrCa) cells.

**Methods:**

CD44 expression was determined with immunohistochemistry (IHC) analysis of a breast cancer tissue microarray (TMA). *In vitro *experiments were performed on a panel of invasive BL-BCa cell lines, by using quantitative polymerase chain reaction (PCR), immunoblotting, protease activity assays, and invasion assays to characterize the basis of HA-induced, CD44-mediated invasion.

**Results:**

Expression of the hyaluronan (HA) receptor CD44 associated with the basal-like subgroup in a cohort of 141 breast tumor specimens (*P *= 0.018). Highly invasive cells of the representative BL-BCa cell line, MDA-MB-231 (MDA-MB-231Hi) exhibited increased invasion through a basement membrane matrix (Matrigel) and collagen. In further experiments, HA-induced promotion of CD44 signaling potentiated expression of urokinase plasminogen activator (uPA) and its receptor uPAR, and underpinned an increased cell-associated activity of this serine protease in MDA-MB-231Hi and a further BL-BCa cell line, Hs578T cells. Knockdown of CD44 attenuated both basal and HA-stimulated uPA and uPAR gene expression and uPA activity. Inhibition of uPA activity by using (a) a gene-targeted RNAi or (b) a small-molecule inhibitor of uPA attenuated HA-induced invasion of MDA-MB-231Hi cells through Matrigel. HA/CD44 signaling also was shown to increase invasion of MDA-MB-231 cells through collagen and to potentiate the collagen-degrading activity of MDA-MB-231Hi cells. CD44 signaling was subsequently shown to upregulate expression of two potent collagen-degrading enzymes, the cysteine protease cathepsin K and the matrix metalloprotease MT1-MMP. RNAi- or shRNA-mediated depletion of CD44 in MDA-MB-231Hi cells decreased basal and HA-induced cathepsin K and MT1-MMP expression, reduced the collagen-degrading activity of the cell, and attenuated cell invasion through collagen. Pharmacologic inhibition of cathepsin K or RNAi-mediated depletion of MT1-MMP also attenuated MDA-MB-231Hi cell invasion through collagen.

**Conclusion:**

HA-induced CD44 signaling increases a diverse spectrum of protease activity to facilitate the invasion associated with BL-BCa cells, providing new insights into the molecular basis of CD44-promoted invasion.

## Introduction

Breast cancer is a heterogeneous disease, currently defined as a minimum of five distinct molecular subtypes [1]. Of these subtypes, "basal-like" breast cancer (BL-BCa) has the worst clinical outcome and is associated with an increased risk of hematogenous metastasis, predominantly to the lungs and liver [2]. An enhanced understanding of the mechanisms and factors that underpin the local invasion and the capacity of BL-BCa cells to escape from the primary tumor, or invade secondary tumor sites, would have significant impact on improving the outcomes for this disease subtype.

Hyaluronan (HA) is a constituent of extracellular matrix, which can induce marked effects on cell behavior by binding to its predominant cell-surface receptor CD44 [3]. Before the era and definition of the molecular subtypes, elevated levels of HA in tumor stroma were shown to correlate with poorly differentiated tumors, auxiliary lymph node status, and short overall survival in breast cancer [4,5]. Klingbeil and colleagues [6] recently determined that CD44 expression associates with the BL-BCa subtype. Furthermore, we recently determined that CD44 is inversely associated with estrogen receptor (ER) expression, with strong expression localized to basal cells [McFarlane S, Conlon S, O'Grady A, Kay EW, Waugh DJJ, unpublished observations]. Consistent with an association with the most clinically aggressive tumors, *in vitro *studies have demonstrated the role of HA and CD44 in stimulating breast cancer cell migration and cell invasion. We have shown that tetracycline-induced expression of CD44 in the noninvasive, luminal MCF-7 breast cancer cell line is alone sufficient to induce cell invasion in response to HA *in vitro *[7]. The induction of CD44 also was sufficient to promote the spontaneous metastasis of these noninvasive luminal breast cancer cells to the liver *in vivo *[8]. Clinical studies have also confirmed the enrichment of CD44 expression in disseminated tumor cells resident in secondary tissue sites [9,10].

Metastasis demands that cancer cells invade through the physical barriers provided by the extracellular matrix of the primary and secondary tumor sites and the basement membranes present within each of these tissue sites. Activation of proteolytic enzymes is thought to be essential in facilitating the degradation of the proteins that constitute these physical structures. Interestingly, two enzymes of the matrix metalloproteinase (MMP) family, MMP-9 and MMP-7, were previously shown to complex with the ectodomain of CD44 on the surface of malignant cells, suggesting that CD44 acts in a structural capacity to concentrate protease activity on the surface of actively invading cells [11,12]. Moreover, CD44 itself is a substrate of a further MMP, which complexes with the membrane-tethered enzyme MT1-MMP and is then cleaved by the enzyme [13,14]. Although these studies suggest that CD44 cooperates with MMPs to regulate cell invasion, the relation of HA-induced CD44 signaling to the regulation of protease expression and activity in invasive breast cancer cells is poorly defined. Moreover, it is well known that malignant breast cancer cells express other protease species in addition to those of MMPs, including the serine protease urokinase plasminogen activator (uPA), a marker of poor prognosis and associated with BL-BCa [15,16].

The objective of our study was to increase our understanding of CD44-promoted breast cancer cell invasion by defining the effect of CD44 signaling on protease gene expression and activity, and defining the role of these proteases in underpinning HA-induced invasion. Informed by studies on breast cancer tissue reaffirming the association of CD44 with the basal-like subtype of breast cancer, we show that CD44 signaling amplifies serine protease, MMP, and/or cysteine cathepsin gene expression and activity, all of which contribute to the invasion of BL-BCa cells through a specialized matrix. Our studies thus provide a new molecular insight to substantiate the association of CD44 with the metastasis of breast cancer.

## Materials and methods

### Tissue microarray construction and immunohistochemistry

Breast tumor samples were collected and data recorded as described previously [17]. Then 4 μm sections were cut from the TMA and immunostained with anti-CD44 on an automated platform (Bond system; Vision BioSystems, Mount Waverley, Victoria, Australia). In brief, cut sections were subjected to on-board dewaxing (Dewax solution; Vision BioSystems) and antigen retrieval (Epitope Retrieval 1 solution; Vision BioSystems) for 20 minutes before application of primary antibody (1:200) and detection by using the Bond polymer Refine detection system (Vision BioSystems). All sections were counterstained with hematoxylin. Negative controls were included for all sections by the omission of primary antibody. Positive control tissue (normal thymus) was also used. Immunostained slides were scored on the proportion of positive tumor cells (range, 0 to 4) and the average intensity of staining (range, 0 to 3). These values were added to obtain a total score (range, 0 to 7). All scores were examined by two independent observers (SMcF and SC), and 10% were also scored by a third independent observer (EK). An expression correlation analysis between CD44 and the basal-like subtype in breast cancer cells and primary tumors was also performed by using Oncomine (Compendia Bioscience, Ann Arbor, MI, USA).

### Cells

The MDA-MB-157 cell line was purchased from American Type Culture Collection (ATCC) (Manassas, VA, USA) and cultured as previously described [7]. Hs578T cells were provided by Dr Paul Mullan (CCRCB, Queen's University Belfast) and cultured as described [18]. The highly invasive clone, MDA-MB-231Hi, was provided, complete with matched parental cells by Prof. Toshiyuki Yoneda (University of Health Sciences, San Antonio, TX, USA) and were cultured in DMEM supplemented with 10% vol/vol fetal calf serum (FCS) (Invitrogen Life Technologies, Paisley, UK) [19]. CD44-depleted MDA-MB-231 cells (termed MDA-MB-231 sh#1) were generated in our laboratory by stable transfection with a CD44 shRNA; parallel transfection with a nontargeting shRNA was used as a control to generate MDA-MB-231 NT cells (McFarlane S, *et al*., unpublished data) and cultured in DMEM supplemented with 10% vol/vol FCS, 0.2 μg/ml puromycin. All cell lines were grown to 70% confluence before experimentation.

### Reagents and antibodies

Chemicals were supplied by Sigma Chemical Co. (St. Louis, MO, USA) unless otherwise stated. Hyaluronan of molecular mass 220 kDa and medical-grade purity was purchased from Lifecore Biomedical Inc. (Chaska, MN, USA). The cathepsin K inhibitor was purchased from Calbiochem (La Jolla, CA, USA). Pefabloc uPA inhibitor was supplied by DSM Nutritional Products Ltd Branch, Pentapharm (Basel, Switzerland). Specific RNAi SMARTpools for CD44, uPA, and MT1-MMP, the nontargeting siCONTROL RNAi oligonucleotide, and Dharmafect 2 transfection reagent were obtained from Dharmacon (Lafayette, CA, USA). The mouse anti-human CD44 monoclonal antibody (1:500), mouse anti-human uPAR mAb (1:500 dilution), and mouse anti-human serpin E1/PAI-1 mAb (1:500) were obtained from R&D Systems (Abingdon, UK). Abcam (Cambridge, UK) supplied the rabbit anti-cathepsin K pAb (1:100). The mouse anti-human urokinase mAb (1:500) and the mouse anti-human PAI-2 mAb (1:3,000) were provided by American Diagnostica (Stamford CT, USA). The mouse anti-MMP14 (MT1-MMP) mAb (1:1,000) was obtained from Chemicon (Watford, UK). Mouse anti-human GAPDH mAb (1:3,000) was supplied by AbD Serotec (Oxford, UK). Sigma (UK) supplied the mouse anti-human β-tubulin mAb (1:1,000). The sheep anti-mouse IgG/horseradish peroxidase conjugate (1:2,000) and donkey anti-rabbit IgG/horseradish peroxidase conjugate (1:2,000) secondary antibodies were purchased from Amersham (Amersham, UK). The anti-p38 MAPK antibody (1:1,000) was supplied by Cell Signaling Technologies (Beverly, MA, USA).

### Quantitative PCR analysis

RNA was harvested and cDNA was synthesized from 10 to 20 μg total RNA, as previously described [7]. Analysis was performed on a Roche LC480 Light Cycler (Roche Diagnostics GmbH, Mannheim, Germany), with product amplification determined by SYBR Green 1 fluorescence detection. Forward (Fwd) and reverse (Rev) primers are as shown: CD44 Fwd TTTGCATTGCAGTCAACAGTC; CD44 Rev GTTACACCCCAATCTTCATGTCCAC; Cathepsin K Fwd AGGCTTCTCTTGGTGTCCATA; Cathepsin K Rev CCTTTCTTTCGATAGTCGACA; MT1-MMP Fwd AATATGGCTACCTGCCTCCC; MT1-MMP Rev TTGCCATTTGAGACCCTGGAT; uPA Fwd GAGGCCCCGCTTTAAGATTA; uPA Rev TGGAGTTAAGCCTTGAGCGA; uPAR Fwd AAGATCACCAGCCTTACCGA; uPAR Rev CCTTCTTCACCTTCCTGGAT; PAI-1 Fwd CTGACAACAGGAGGAGAAAC; PAI-1 Rev GGAACAGCCTGAAGAAGTGG; PAI-2 Fwd ACCCAGAACCTCTTCCTCTC; PAI-2 Rev TGGTAAAGTTCTCTGGAGTCA; 18S Fwd CATTCGTATTGCGCCGCTA; 18S Reverse CGACGGTATCTGATCGTC. Reactions were conducted by using 1 μl of cDNA reverse transcribed from 10 to 20 μg total RNA, 0.4 μ*M *final concentration of forward and reverse primers and 2 × SYBR Green 1 master mix (Roche Diagnostics). Standard cycling procedures were used, with annealing temperatures of 51°C used for the CD44 and 18S, 54°C for uPAR, 55°C for cathepsin K, 55.7°C for MT1-MMP, and 57°C for the uPA, PAI-1, and PAI-2 primer pairs. Specific amplicon formation with each primer pair was confirmed by melt-curve analysis. Gene expression was quantified relative to an 18S housekeeping gene.

### Knockdown of CD44, uPA, and MT1-MMP expression in cancer cells

Cells were seeded to approximately 70% confluence before transfection with the relevant RNAi SMARTPool or the nontargeting RNAi oligonucleotide by using DharmaFECT-2 according to the manufacturer's instructions.

### Immunoblotting

Protein samples were collected, quantified, and blotted as previously described [20] by using the antibodies described. Immunoreactivity was detected by using chemiluminescence (Supersignal; Pierce). Equal loading of the protein samples was assessed by reprobing the membrane with either a GAPDH or β-tubulin antibody.

### uPAR flow cytometry

Samples were analyzed for cell-surface uPAR expression as previously described [21] by using a fluorescein-conjugated murine anti-human monoclonal uPAR antibody (American Diagnostica Inc., Stamford, CT, USA) and an IgG control antibody (R&D Systems, Abingdon, UK).

### Invasion assays

Invasion chambers were prepared by coating cell-culture inserts (12-μm pore size; Costar) with either 2 μg/cm^2 ^of Collagen I (BD Biosciences, Erembodegem, Belgium) or 100 μg/cm^2 ^of Matrigel (BD Biosciences) alone or supplemented with HA at a final concentration of 100 μg/cm^2 ^in phenol red-free DMEM. Assays were conducted as previously described [7]. In some experiments, the invasive capacity of the cells was determined by using the xCELLigence cell analyzer system (Roche Diagnostics). The top chambers of CIM invasion plates were coated with Matrigel (5%) and allowed to dry for 4 hours at 37°C. Prewarmed phenol red-free DMEM supplemented with FCS was added to the lower chambers before the upper chamber was locked into place and 30-μl serum-free medium added to each well. The invasion apparatus was allowed to equilibrate at 37°C for 1 hour, after which 1 × 10^5 ^cells in serum-free medium were seeded into the top wells and allowed to settle for 30 minutes. The plates were then loaded onto the xCELLigence analyzer, and electrical impedance was measured every 15 minutes over a 36-hour period.

### uPA activity assays

uPA activity was determined by using a uPA Activity Assay Kit (Chemicon International) according to the manufacturer's instructions on either cell lysate or cell media samples. In brief, a standard curve was generated by the addition of varying amounts of the uPA-positive control (10 to 160 μl) in duplicate to a clear 96-well plate. Then 50μl cell lysate samples or 10 μl of cell media samples was added to the 96-well plate along with a either a media or RIPA buffer blank. Sufficient deionized water was added to bring the total volume of each well to 160 μl. Then 20 μl assay buffer was added to each well, followed by 20 μl of chromogenic substrate. The plate was incubated in the dark at 37**°**C for periods between 10 minutes and 8 hours, depending on uPA levels contained in samples. The optical density of each well was determined by using a standard microplate reader (405 nm). Duplicate OD readings for each sample were averaged, and the relevant blanks were subtracted. Samples were compared with the standard curve to obtain relative uPA activity. Results are expressed as fold change over control cells.

### Plasmin activity assays

Cells were plated at a density of 2 × 10^4 ^cells per well in 200 μl of phenol red-free medium and allowed to reach approximately 70% confluence. Cell-free medium (200 μl) was added to a well as a negative control, and 3 μg human plasminogen (Hyphen BioMed, ZAC Neuville University, France) was added to each well and incubated at 37°C for 30 minutes, and 65 μl of assay buffer (100 m*M *Tris/0.5% TritonX-100, pH 8.8) and 15 μl S-2251 substrate (25 μg) (Chromogenix, Milan, Italy) was added to the wells of a separate 96-well plate. After a 30-minute incubation period, 20 μl of each sample was added in duplicate to wells. Human plasmin was implemented to generate a standard curve, from which plasminogen activation rates of unknown samples were determined. Plasmin of varying amounts was added to the wells of the 96-well plate containing assay buffer and S-2251 substrate. The plate was covered, and the optical density for each well at 405 nm was determined by using a standard microplate reader at regular intervals. Plasminogen is activated to form plasmin that is able to cleave the chromogenic substrate S-2251. The method for determination of activity is based on the difference in absorbance at 405 nm. Duplicate OD readings for each sample were averaged, and the mean media blank subtracted. Samples were compared with the plasmin standard curve to obtain relative plasmin units of activity. Results were then quantified relative to cell number for each sample, and expressed as relative plasmin units of activity per 1 × 10^6 ^cells.

### Cathepsin K ELISA

Breast cancer cells were seeded at a density of 1 × 10^5 ^cells/500 μl in a 24-well plate and allowed to reach 70% confluence before experimentation. Cell-culture supernatants were collected and cell debris removed by centrifugation. Cell counts were taken from each experimental well to allow normalization of cathepsin K concentration to cell number (10^6 ^cells). The cathepsin K ELISA (Biomedica Gruppe, Vienna, Austria) was conducted as per the manufacturer's instructions. In brief, 50 μl of the sample, standards or cathepsin K control were added to the polyclonal sheep anti-cathepsin K-coated microtiterstrips and incubated at room temperature for 20 to 24 hours in the dark. The samples, standards, and control were aspirated and wells washed 5 times with wash buffer. The 200-μl substrate was added to each well and incubated for 30 minutes at room temperature in the dark. The 50-μl STOP solution was added to each well, and the absorbance was immediately read. The sample concentration was calculated from the standard curve, and the concentration normalized to 10^6 ^cells by using previously determined cell counts.

### EnzChek collagen degradation assay

Collagenase activity was measured by using the EnzChek Gelatinase/Collagenase assay kit (Molecular Probes, Eugene, OR, USA) according to the manufacturer's instruction. In brief, cells were grown in P90s in serum-containing media and allowed to reach 80% confluence before concentration of the cell culture media by using Centriplus centrifugal filter units (Millipore, Billerica, MA, USA). The concentrated protein samples were quantified by using the BCA assay, and 1 mg protein was loaded in duplicate onto a 96-well black fluorescence plate. Then 20 μl of DQ Collagen type I fluorescein conjugate (Molecular Probes) was loaded into each well along with 80 μl 1 × Running Buffer. The collagenase *Clostridium *(0.025 U/ml) was run as a positive control and running buffer acted as a negative control. As an additional control, cell-culture media (in the absence of cells) was assessed to ensure that the collagenase activity was solely restricted to enzymes secreted by the breast cancer cells. The fluorescence intensity was read at 37°C in a fluorescence microplate reader (Cytofluor 4000; Applied Biosystems, Warrington, UK) over a 6- to 8-hour period with fluorescent readings taken every 30 minutes. Collagenase activity over the time period was reported as fluorescence on subtraction of the cell-culture media fluorescence.

### Statistical analysis of experimental data

Differences between data points (invasion and enzyme activity assays, ELISA experiments) were assessed for statistical significance by using the two-tailed Student *t *test comparisons (GraphPad Prism 5 software).

## Results

### CD44 expression correlates with triple-negative, basal-like breast cancers

CD44 expression was assessed in a breast cancer tissue microarray composed of tissue cores extracted from a total of 141 patients, representing all molecular subtypes of the disease. ER was expressed by 86.7% (121 of 140) of the cases, and PR was expressed in 69% (96 of 140) of cases. Expression of CD44 was detected in 60% (84 of 141) of the cases, with strong expression detected in 46% (65 of 141) of the cases analyzed. A strong inverse correlation between CD44 expression and ER status (*P *= 0.025) and PR status (*P *= 0.021) was found, but no correlation was found to Her2 expression. Instead, CD44 expression was strongly correlated with the basal-like phenotype (*P *= 0.018) and the triple-negative subtype (*P *= 0.018) (Table 1).

**Table 1:** Association of CD44 with molecular characteristics of breast tumors

	Association of CD44 expression with tumor characteristics					
			***n *(%)**	**CD44 *n *(%)**	***P***	

	CD44	+	84/141 (60%)	N/A		
		-	57/141 (40%)	N/A		
	ER	+	121/140 (86%)	73/121 (60%)	0.025 Mann-Whitney, 0.019 *t *test	
		-	19/140 (14%)	16/19 (84%)		
	PR	+	96/140 (69%)	56/96 (58%)	0.021 Mann-Whitney, 0.025 *t *test	
		-	44/140 (31%)	33/44 (75%)		
	Her2	+	13/133 (10%)	75/120 (63%)	Not significant	
		-	120/133 (90%)	8/13 (62%)		
	Basal	+	15/135 (11%)	13/15 (87%)	0.018 Mann-Whitney, 0.021 *t *test	
		-	120/135 (89%)	72/120 (60%)		
	Triple negative	+	15/135 (11%)	13/15 (87%)	0.018 Mann-Whitney, 0.021 *t *test	
		-	120/135 (89%)	72/120 (60%)		
	Expression of CD44 is expressed as a percentage of patients in individual clinical classifications (first column).					

**Figure 1:**
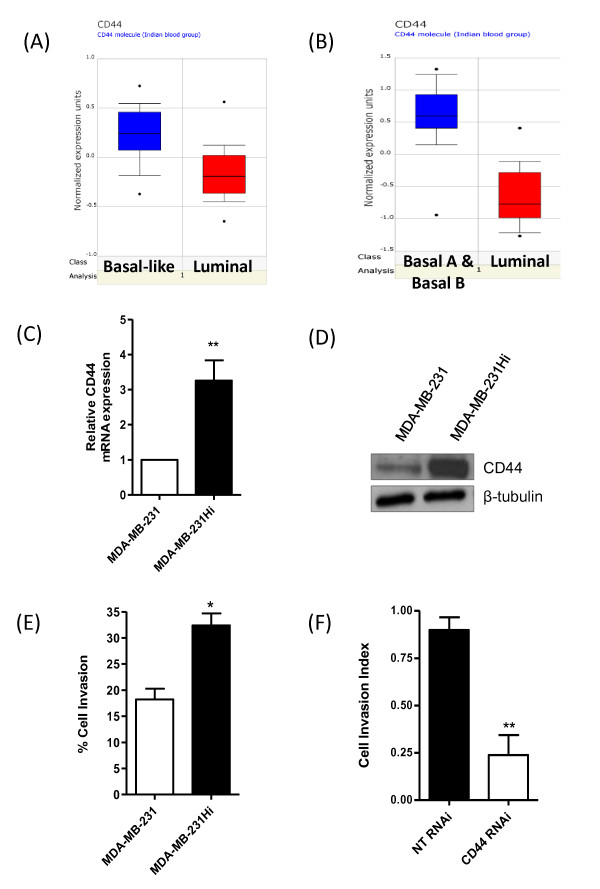
**Association of CD44 with highly aggressive breast tumor subtypes or highly invasive breast cancer cells**. Oncomine analysis of CD44 expression in breast cancer demonstrated significantly higher CD44 mRNA expression in **(A) **basal-like breast cancer tumors in comparison with luminal tumors and in **(B) **basal-like breast cancer cells lines in comparison with luminal-like cell lines (*P *= 1.90E-6). **(C) **Bar graph showing elevated CD44 mRNA transcript levels present in the MDA-MB-231Hi cells compared with the MDA-MB-231 parental cells (3.26-fold increase; *n *= 5; *P *< 0.01). **(D) **Representative immunoblot showing upregulation of CD44s in MDA-MB-231Hi cells relative to the MDA-MB-231 parental cell line. The blots were reprobed with β-tubulin as a loading control. **(E) **Bar graph illustrating the elevated invasive capacity of the MDA-MB-231Hi cells (32.28% ± 2.33% of invaded cells/total number of cells; *n *= 3; *P *< 0.05) through Matrigel relative to the MDA-MB-231 cells (18.23% ± 2.071%). **(F) **CD44 knockdown in MDA-MB-231Hi reduced the cell-invasion index over a 36-hour period through Matrigel from 0.89 ± 0.07 to 0.24 ± 0.11 (*n *= 3; *P *< 0.01). Statistically significant differences were determined by using a Student two-tailed *t *test (*P < 0.05; **P < 0.01).

To validate these results further, we performed an Oncomine analysis of two publically available breast cancer datasets [18,22]. Expression of CD44 was strongly associated with basal-like tumors (Figure 1A). Moreover, the expression of CD44 was preferentially distributed to basal-like cell lines, as opposed to luminal cell lines (Figure 1B).

### Characterization of increased CD44 expression and importance to invasion in basal-like breast cancer cells

Given the strong correlation of CD44 expression to the basal-like subgroup of breast cancers, a series of *in vitro *experiments was performed by using cell lines representative of this subtype, primarily by using the invasive MDA-MB-231 cell line and a highly metastatic derivative MDA-MB-231 cell line (MDA-MB-231Hi). After qPCR and immunoblotting analysis, we confirmed that MDA-MB-231Hi cells were heavily enriched for CD44 at both mRNA (Figure 1C) and protein level (Figure 1D) when compared with their parental MDA-MB-231 counterparts, and were more invasive through Matrigel (Figure 1E). The importance of CD44 and its elevated expression in underpinning the more-efficient invasion of MDA-MB-231Hi cells was confirmed by using an RNAi strategy targeting this cell-surface receptor; knockdown of CD44 (validated in Additional file 1, Figure S1A) significantly reduced invasion of the MDA-MB-231Hi cells through Matrigel (Figure 1F).

### Characterization of elevated uPA activity in highly invasive basal-like breast cancer cells

**Figure 2:**
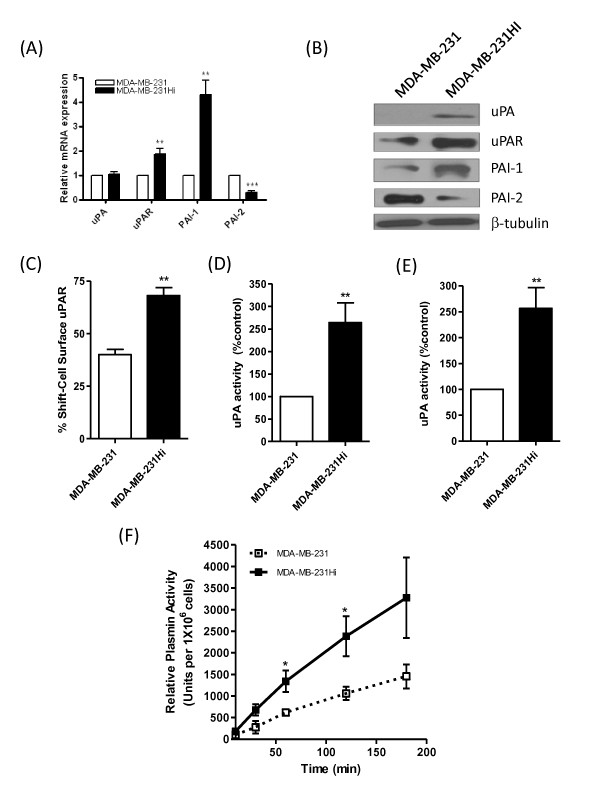
**Characterization of elevated expression or activity of the uPA signaling pathway in highly invasive breast cancer cells**. **(A) **Bar graph showing the comparable levels of mRNA transcript expression for uPA, uPAR, and its endogenous inhibitors PAI-1 and PAI-2 between MDA-MB-231Hi and parental MDA-MB-231 cells. **(B) **Immunoblots showing the elevated uPA, uPAR, and PAI-1 expression and decreased PAI-2 expression in MDA-MB-231Hi cells relative to MDA-MB-231 cells. The blots were reprobed with β-tubulin as a loading control. **(C) **Flow cytometry confirming increased cell-surface uPAR expression in MDA-MB-231Hi cells (68.08% ± 3.770% shift relative to 40% ± 2.492% in MDA-MB-231 cells; *P *< 0.01; *n *= 3). Next, activity assays were used to detect changes in uPA activity, measured within a "cell-associated" fraction **(D) **and within the supernatant **(E) **of the cell culture. Activity of this serine protease was significantly higher in both the cell-associated fraction (2.64-fold increase; *P *< 0.01; *n *= 4) and the supernatant (2.56-fold increase; *P *< 0.01; *n *= 4) of the MDA-MB-231Hi cells compared with the parental MDA-MB-231 cells. **(F) **An increased level of plasmin activity was observed in the MDA-MB-231Hi cells relative to parental cells, conducted over a 3-hour period (*P *< 0.05; *n *= 4). Statistically significant differences in all quantitative assays were determined by using a Student two-tailed *t *test (*P < 0.05; **P < 0.01; ***P < 0.001).

uPA is a serine protease associated with the invasion and intravasation of cancer cells [23-25]. The expressions of uPA, its receptor uPAR, and its endogenous inhibitors PAI-1 and PAI-2 were characterized at mRNA and protein levels. Transcript levels for uPAR and PAI-1 were elevated in the more-invasive MDA-MB-231Hi cells, whereas PAI-2 expression was decreased relative to MDA-MB-231 parental cells. No change was noted in uPA mRNA levels (Figure 2A). Immunoblotting experiments, however, confirmed an increase in uPA, uPAR, and PAI-1 expression in the more-invasive MDA-MB-231Hi cells, whereas PAI-2 expression was again decreased relative to MDA-MB-231 cells (Figure 2B). Flow-cytometry analysis confirmed an increased level of cell-surface expression of uPAR in the MDA-MB-231Hi cells relative to MDA-MB-231 cells (Figure 2C). Activity assays were used to detect changes in uPA activity, measured within a "cell-associated" fraction and within the supernatant of the cell culture. Activity of this serine protease was significantly higher in both the cell-associated fraction (Figure 2D) and the supernatant (Figure 2E) of the MDA-MB-231Hi cells when compared with the parental MDA-MB-231 cell line. Consistent with increased uPA secretion in the supernatant fraction, we also detected an increased level of plasmin activity in the supernatant of the MDA-MB-231Hi cells relative to the plasmin activity observed in the MDA-MB-231 cells, measured over a 3-hour period (Figure 2F).

### HA and CD44 regulate uPA expression and activity in basal-like breast cancer cells

**Figure 3:**
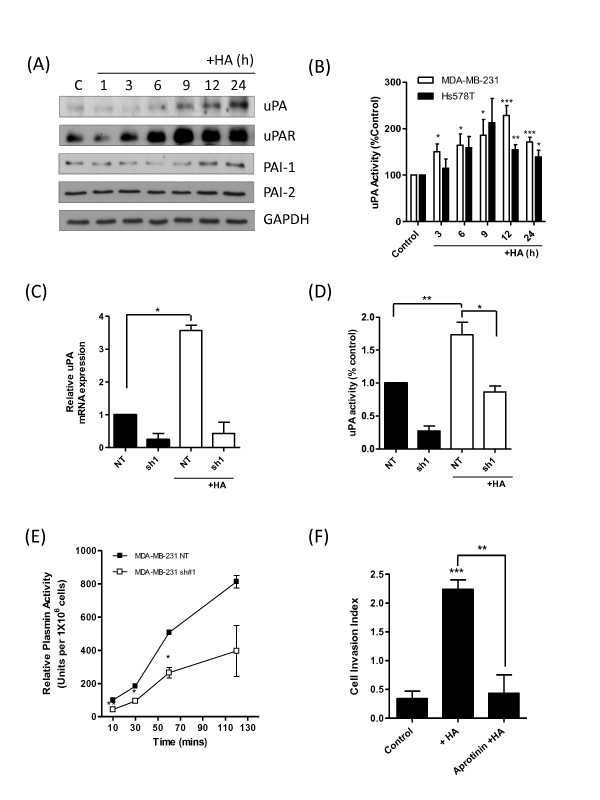
**CD44 signaling potentiates the expression and activity of uPA-signaling in breast cancer cells**. **(A) **Immunoblot showing enhanced uPA, uPAR, and PAI-1 protein expression on HA stimulation (100 μg/ml) of MDA-MB-231 cells. No change in PAI-2 protein expression was found. All immunoblots shown were reprobed with GAPDH as a loading control. **(B) **Bar graph presenting time-dependent increases in cell surface-associated uPA activity detected in MDA-MB-231 and Hs578 BL-BCa cells in response to exogenous HA stimulation (100 μg/ml). **(C) **Bar graph comparing the mRNA transcript levels for uPA in MDA-MB-231 NT and MDA-MB-231 sh#1 cells, in the absence and presence of an exogenous HA stimulus (100 μg/ml). **(D) **Bar graph comparing the cell surface-associated uPA activity in MDA-MB-231 NT and MDA-MB-231 sh#1 cells, in the absence and presence of an exogenous HA stimulus (100 μg/ml). **(E) **Bar graph comparing plasmin activity in MDA-MB-231 NT and MDA-MB-231 sh#1 cells; the loss of CD44 was coupled with a decrease in plasmin activity in these BL-BCa cells (*P *< 0.05; *n *= 3). **(E) **Invasion assays were conducted in HA-supplemented Matrigel in the presence or absence of aprotinin for a period of 36 hours. HA increased the cell-invasion index of MDA-MB-231Hi cells from 0.3392 ± 0.1297 to 2.237 ± 0.1659 (*P *< 0.001). In the presence of 1 μ*M *aprotinin, the cell-invasion index was reduced to 0.4312 ± 0.3191 (*P *< 0.01 relative to HA alone; *n *= 3). Statistically significant differences in quantitative values were determined by using a Student two-tailed *t *test (*P < 0.05; **P < 0.01; ***P < 0.001).

We next sought to determine the association of HA and CD44 to increased uPA expression and activity in BL-BCa cells. Initially, MDA-MB-231 cells were stimulated with HA to determine whether the native ligand of CD44 induced expression of uPA or that of its associated proteins. Immunoblotting analysis confirmed that exogenous HA increased the expression of uPA, uPAR, and PAI-1, without affecting the expression of PAI-2 (Figure 3A); marked increases in uPA and uPAR expression were detected 9 hours after stimulation with HA. Furthermore, qPCR analysis also revealed that exogenous HA increased mRNA transcript levels for these genes in a second BL-BCa cell line, Hs578T. Significant increases in uPA mRNA transcript levels were prominent in Hs578T cells for 24 hours afterstimulation with HA, whereas mRNA transcript levels for uPAR and PAI-1 were increased relative to those detected for these genes in unstimulated cells (see Additional file 2, Figure S2). Consistent with our observations of increased gene and protein expression in response to exogenous HA, stimulation of MDA-MB-231 and Hs578T cells with HA was shown to increase cell surface-associated uPA activity in both of these BL-BCa cell lines (Figure 3B).

The role of CD44 in regulating uPA and its associated proteins was next investigated. A short-hairpin strategy was used to downregulate CD44 mRNA and protein expression in the MDA-MB-231Hi cell line (termed MDA-MB-231 sh#1 cells). The effects of CD44 knockdown were determined by comparison with MDA-MB-231 NT cells, transfected with a nontargeting short hairpin and which retained CD44 expression (see Additional file 3, Figures S3A and S3B). CD44 knockdown retarded cell invasion through Matrigel (see Additional file 3, Figure S3C). Relative to a nontargeting short hairpin (NT), loss of CD44 also coincided with a decrease in the transcript levels for uPA, uPAR, PAI-1, and PAI-2 (all *P *< 0.001) (see Additional file 3, Figure S3D) and a decreased expression for each of these proteins (see Additional file 3, Figure S3E).

To associate CD44 further in mediating HA-induced increases in uPA gene expression and activity, we performed another series of experiments using the MDA-MB-231 sh#1 cells. In these experiments, the effect of CD44 knockdown on the expression and activity of uPA was determined in the absence and presence of an exogenous HA stimulus. Loss of CD44 not only coincided with a decreased transcript level of uPA relative to that in NT-transfected cells, as observed before, but also attenuated the HA-promoted increases in uPA gene expression (Figure 3C). When further experiments were conducted to measure uPA activity, MDA-MB-231 sh#1 cells had reduced cell-surface uPA activity under unstimulated conditions, whereas the HA-induced increase in uPA activity was severely truncated in the absence of CD44 (Figure 3D).

Consistent with the observed decreases in uPA and uPAR mRNA and protein expression, and the reduction in uPA activity, the loss of CD44 was also shown to correlate with a decrease in the plasmin activity associated with the MDA-MB-231Hi cells (Figure 3E). The functional relevance of this uPA-generated plasmin activity to HA-promoted cell invasion was determined with a further invasion assay in which plasmin activity was inhibited by using aprotinin. HA failed to stimulate invasion in the presence of 1 μ*M *aprotinin (Figure 3F).

### uPA activity underpins HA-promoted invasion of basal-like breast cancer cells

**Figure 4:**
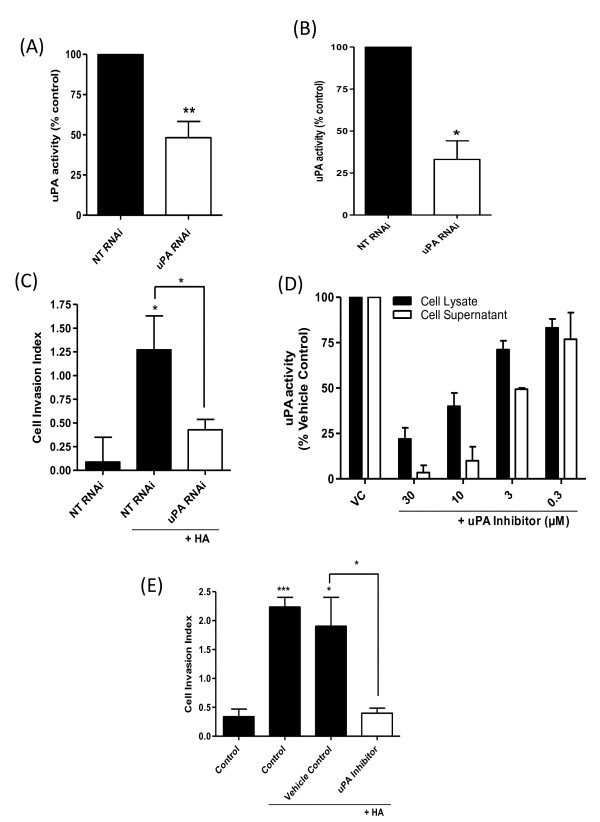
**Downregulation of uPA activity attenuates the HA-promoted invasion of CD44-enriched invasive breast cancer cells**. RNAi-mediated reduction of uPA expression in MDA-MB-231Hi cells resulted in a decrease in **(A) **cell-surface uPA activity to 64.9% ± 10.8% of control NT RNAi-transfected cells (*P *< 0.01; *n *= 3) and **(B) **secreted uPA to 33.1% ± 11.1% of control NT RNAi-transfected cells (*P *< 0.05; *n *= 3). **(C) **Bar graph illustrating the HA-promoted invasion of MDA-MB-231 NT cells through Matrigel over a 12-hour period, in the absence and presence of a uPA-targeting siRNA. HA (100 μg/ml) increased the cell-invasion index to 1.27 ± 0.36 (*P *< 0.05); however, this was attenuated by transfection with the uPA RNAi SMARTPool (cell invasion index of 0.43 ± 0.1; *P *< 0.05 relative to HA alone; *n *= 4). **(D) **Bar graph presenting the effects of administering an uPA inhibitor on uPA activity detected in MDA-MB-231Hi cells. **(E) **Bar graph illustrating the HA-promoted invasion of MDA-MB-231Hi cells in the absence and presence of a uPA inhibitor. HA increased MDA-MB-231Hi cell invasion through Matrigel over a 36-hour period to a mean cell index of 2.24 ± 0.2 (*P *< 0.001 relative to control cells). Treatment with the uPA inhibitor (30 μ*M*) reduced the HA-promoted cell-invasion index to 0.4 ± 0.09 (*P *< 0.05 relative to vehicle control; *n *= 3). Statistically significant differences were determined by using a Student two-tailed *t *test (*P < 0.05; **P < 0.01; ***P < 0.001).

A further series of experiments was conducted to determine the importance of uPA in HA-promoted invasion. Expression of uPA was inhibited by using RNAi. Transfection with three increasing concentrations of uPA-targeting oligonucleotides decreased protein expression of uPA in the MDA-MB-231Hi cells (see Additional file 4, Figure S4A). Transfection with 200 n*M *uPA RNAi significantly reduced transcript levels for uPA in the MDA-MB-231Hi cells (see Additional file 4, Figure S4B) and significantly decreased cell-surface associated and secreted uPA activity in these cells (Figure 4A and 4B). Furthermore, uPA knockdown significantly reduced the invasiveness of MDA-MB-231Hi cells through Matrigel (Figure 4C).

A pharmacologic approach used a small-molecule uPA inhibitor to attenuate uPA activity. Administration of this inhibitor to MDA-MB-231Hi cells reduced both cell surface-associated uPA activity and uPA activity in the supernatant in a concentration-dependent manner; at a concentration of 30 μ*M*, this inhibitor reduced supernatant activity to <5% of vehicle-treated control and reduced cell surface-associated activity to 22 ± 6.1% of vehicle-treated control (Figure 4D). In cell-invasion assays, the addition of the uPA inhibitor (30 μ*M*) abrogated the HA-stimulated invasion of the MDA-MB-231Hi cells (Figure 4E).

### CD44 signaling also increases expression of collagenolytic proteases of the cysteine cathepsin and matrix-metalloproteinase families in basal-like breast cancer cells

**Figure 5:**
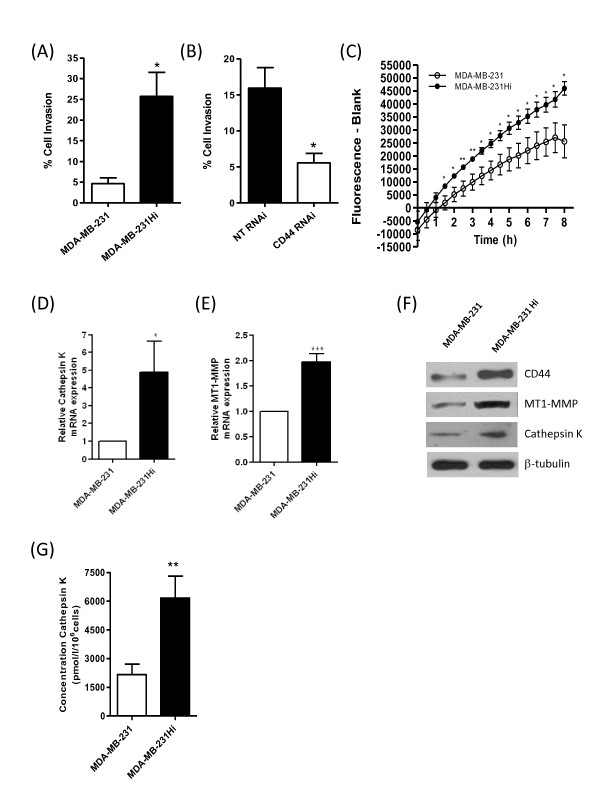
**Highly invasive breast cancer cells exhibit increased expression of collagen-degrading enzymes**. **(A) **Bar graph illustrating the comparable invasion of parental MDA-MB-231 and MDA-MB-231Hi cells through a collagen I matrix. **(B) **Bar graph illustrating the retardation of MDA-MB-231Hi cell invasion through collagen I after transfection of the cells with a CD44 RNAi SMARTPool. **(C) **Bar graph presenting the respective collagenolytic activity of MDA-MB-231 parental and MDA-MB-231Hi cells, determined by using the Enzchek Collagenase assay. Bar graphs showing the results of quantitative real-time PCR analysis to determine mRNA transcript expression for **(D) **cathepsin K and **(E) **MT1-MMP in parental MDA-MB-231 and MDA-MB-231Hi cells. MDA-MB-231Hi cells demonstrated a 3.98- and 1.98-fold increase in cathepsin K and MT1-MMP, respectively, compared with their parental counterparts. **(F) **Immunoblot demonstrating elevated CD44, MT1-MMP, and cathepsin K protein expression in MDA-MB-231Hi cells relative to MDA-MB-231 parental cells. Membranes were reprobed with β-tubulin as a loading control. **(G) **Enzyme-linked immunosorbent assay (ELISA) showing elevated cathepsin K secretion in the MDA-MB-231Hi cells (6,170 ± 1,148 pmol/L/10^6 ^cells) relative to MDA-MB-231 parental cells (2,164 ± 553.3 pmol/L/10^6 ^cells; *P *< 0.01; *n *= 6). Statistically significant differences were determined by using a Student two-tailed *t *test (*P < 0.05; **P < 0.01; ***P < 0.001)

MDA-MB-231Hi cells were also observed to be more invasive on a collagen I matrix relative to the parental MDA-MB-231 cells (Figure 5A). Interestingly, MDA-MB-231Hi cell invasion through collagen I was attenuated by RNAi-mediated knockdown of CD44 (Figure 5B). To determine whether this increased invasion through collagen I was representative of a further difference in protease activity, an *in vitro *fluorescence-based collagenolytic activity assay (EnzChek Gelatinase/Collagenase assay; Molecular Probes) was used. Concentrated cell-culture supernatants from MDA-MB-231 parental and MDA-MB-231Hi cells were incubated with a DQ fluorescein-conjugated collagen I substrate, and fluorescence was read at 30-minute intervals over an 8-hour period. MDA-MB-231Hi cells demonstrated an increased ability to degrade collagen I when compared with MDA-MB-231 parental cells (Figure 5C).

uPA has no intrinsic ability to degrade collagen. Therefore, we undertook experiments to identify additional differentially expressed proteolytic species that can directly promote collagen degradation and whose altered expression/activity may explain the marked difference in cell invasion through collagen. Two of the most potent collagen-degrading proteases are the membrane-tethered matrix metalloproteinase MT1-MMP [26] and the cysteine protease, cathepsin K [27]. qPCR analysis demonstrated an increased mRNA transcript level for cathepsin K (Figure 5D) and MT1-MMP in MDA-MB-231Hi cells over parental cells (Figure 5E). Immunoblotting also confirmed an elevated expression of MT1-MMP and cathepsin K protein expression in the MDA-MB-231Hi cells (Figure 5F), whereas an ELISA revealed an increased secretion of cathepsin K in MDA-MB-231Hi cells relative to parental cells (Figure 5G).

**Figure 6:**
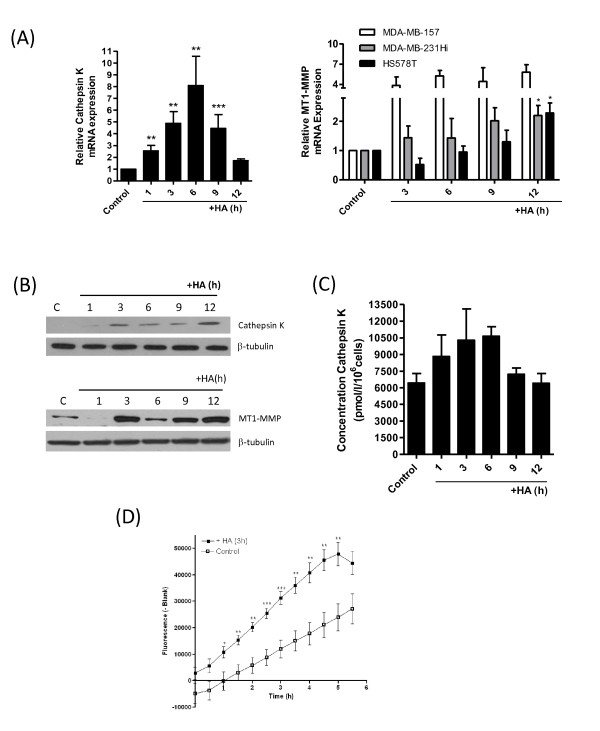
**Characterization of HA-induced potentiation of collagen-degrading enzymes and their role in underpinning CD44-mediated invasion of breast cancer cells**. **(A) **Bar graphs showing the induction of cathepsin K (left panel) and MT1-MMP (right panel) mRNA expression after treatment with 100 μg/ml HA over a 12-hour period. All values were normalized against 18S mRNA expression, and fold-changes were calculated by comparison of mRNA levels in the absence of HA stimulation. Data shown is the mean ± SEM fold-change relative to control determined from three independent experiments. **(B) **A series of immunoblots confirming a time-dependent increase in the expression of cathepsin K (top panel) and MT1-MMP (bottom panel) expression in response to HA-stimulation (100 μg/ml). Equal protein loading in immunoblots was confirmed by reprobing the membranes for β-tubulin. **(C) **ELISA experiments conducted on MDA-MB-231Hi cells on 100 μg/ml HA stimulation demonstrate a time-dependent increase in cathepsin K secretion from 6,437 ± 855.0 pmol/L/10^6 ^cells to 10,649 ± 860.5 pmol/L/10^6 ^cells within 6 hours of HA treatment. Data points are the mean concentration of cathepsin K ± SEM relative to concentration of cathepsin K in untreated MDA-MB-231Hi cells. **(D) **Stimulation of MDA-MB-231Hi cells with HA (100 μg/ml) for 3 hours potentiates the extracellular collagenolytic activity of MDA-MB-231Hi cells by using the *in vitro *EnzChek Collagenase assay (*P *< 0.001; *n *= 5).

The role of HA in regulating the expression of these proteases in invasive BL-BCa cells was studied in further experiments. Addition of exogenous HA induced time-dependent increases in MT1-MMP and cathepsin K mRNA transcript levels (Figure 6A) and increased expression of both proteases (Figure 6B) in MDA-MB-231Hi cells. HA-promoted increases in MT1-MMP gene expression was also observed in two further CD44-expressing BL-BCa cell lines, MDA-MB-157 and Hs578T (Figure 6B), whereas increases in cathepsin K transcript levels were detected in MDA-MB-157 cells (see Additional file 5, Figure S5). Furthermore, addition of HA also potentiated cathepsin K secretion in MDA-MB-231HI cells (Figure 6C). Consistent with the HA-induced increases in cathepsin K and MT1-MMP expression, stimulation of the MDA-MB-231Hi cells for 3 hours with HA increased the rate of increase and the maximal collagenolytic activity exerted by the MDA-MB-231HI cells by using the EnzChek Gelatinase/Collagenase assay (Figure 6D).

**Figure 7:**
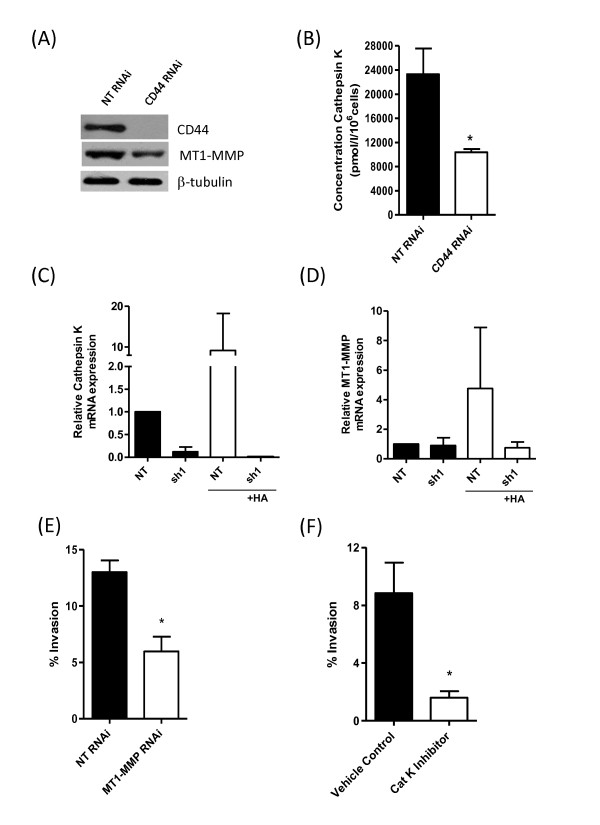
**CD44 regulates MT1-MMP and cathepsin K expression to underpin BL-BCa cell invasion**. **(A) **Immunoblot showing reduced MT1-MMP protein expression on transfection of MDA-MB-231Hi cells with a CD44-targeted RNAi SMARTPool relative to a nontargeting oligonucleotide-transfected cell population. **(B) **Bar graph demonstrating the impact of CD44 knockdown on the secretion of cathepsin K by MDA-MB-231Hi cells. Cathepsin K concentration was reduced from 23,310 ± 4,227 pmol/L/10^6 ^cells in NT cells to 10,380 ± 526.7 pmol/L/10^6 ^cells in CD44-depleted cells (*P *< 0.05; *n *= 4). **(C) **Bar graph comparing the mRNA transcript levels for cathepsin K in MDA-MB-231 NT and MDA-MB-231 sh#1 cells, in the absence and presence of an exogenous HA stimulus (100 μg/ml). **(D) **Bar graph comparing the mRNA transcript levels for MT1-MMP in MDA-MB-231 NT and MDA-MB-231 sh#1 cells, in the absence and presence of an exogenous HA stimulus (100 μg/ml). **(E) **Bar graph illustrating the effect of RNAi-mediated suppression of MT1-MMP in MDA-MB-231Hi cells on their invasion through collagen I. **(F) **Bar graph showing the effect of administering a pharmacologic inhibitor of cathepsin K on the invasion of MDA-MB-231Hi cells through a collagen I matrix. Statistically significant differences between data points in quantitative assays were determined by using a Student two-tailed *t *test. *P < 0.05; **P < 0.01; ***P < 0.001.

Further experiments were conducted to confirm the importance of CD44 in mediating the HA-induced increases in the expression of these collagen-degrading enzymes. Initially, RNAi-mediated depletion of CD44 expression in MDA-MB-231Hi cells was shown to result in a reduced expression of MT1-MMP (Figure 7A) and significantly decreased cathepsin K secretion (Figure 7B). In a further series of experiments, the knockdown of CD44 in the MDA-MB-231 sh#1 cells was shown to reduce mRNA transcript levels for cathepsin K (Figure 7C) and MT1-MMP (Figure 7D). Moreover, the presence of exogenous HA was unable to induce the expression of either of these proteases in the MDA-MB-231 sh#1 cells that had been rendered devoid of CD44 expression, in contrast to the positive change in protease gene expression observed in the MDA-MB-231 NT cells in response to HA (Figure 7C and 7D).

The functional importance of MT1-MMP and cathepsin K in underpinning invasion of these cells through a collagen-I matrix was then investigated by using molecular or pharmacologic approaches. RNAi was used to suppress MT1-MMP expression in the MDA-MB-231Hi cells (see Additional file 1, Figure S1B), whereas a commercially available pharmacologic inhibitor was used to antagonize cathepsin K activity in these cells. Inhibition of MT1-MMP (Figure 7E) or cathepsin K (Figure 7F) reduced the invasive capacity of these MDA-MB-231Hi cells through collagen.

### HA induces transcriptional regulation of proteases through a p38 mitogen-activated protein kinase pathway

**Figure 8:**
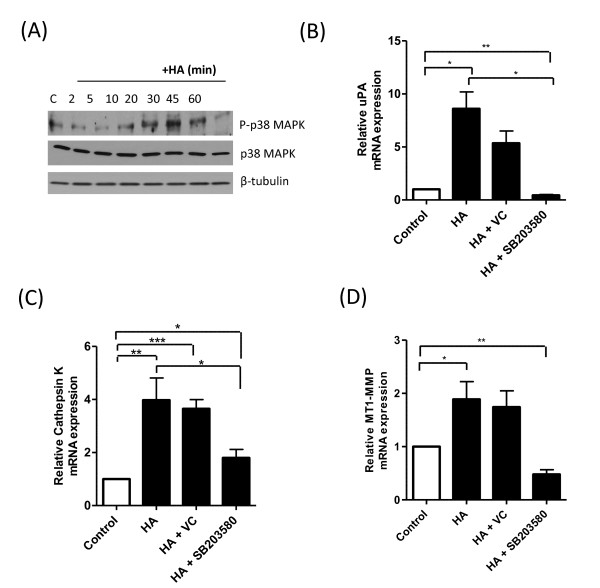
**HA-induced regulation of protease gene expression is attenuated by a p38 MAPK inhibitor**. **(A) **Representative immunoblot characterizing the time-dependent phosphorylation of p38 MAPK in MDA-MB-231Hi cells after stimulation with 100 μg/ml HA. Equal protein loading in immunoblots was confirmed by reprobing the membranes for p38 MAPK and β-tubulin. Bar graphs illustrate the magnitude of HA-induced increases in the mRNA transcript levels for **(B) **uPA, **(C) **cathepsin K, and **(D) **MT1-MMP, in the absence or presence of the p38 MAPK inhibitor SB203580 (2 μ*M*). The effect of the DMSO vehicle on the response was adjusted. Data shown are the mean ± SEM values calculated from three independent experiments. Statistically significant differences between data points in quantitative assays were determined by using a Student two-tailed *t *test. *P < 0.05; **P < 0.01.

HA previously was shown to regulate protease expression in chondrocytes and osteoclasts through induction of p38 mitogen-activated protein kinase (MAPK) [28,29]. Experiments were first conducted to determine whether exogenous HA could induce this signaling pathway in BL-BCa cells. Immunoblotting experiments initially proved that the addition of HA induced a time-dependent phosphorylation of p38 MAPK in MDA-MB-231Hi cells (Figure 8a). The significance of p38 MAPK in underpinning HA-induced transcription of the proteases uPA, cathepsin K, and MT1-MMP in BL-BCa cells was studied further. MDA-MB-231Hi cells were stimulated for 9 hours with HA in the absence or presence of the p38 MAPK inhibitor SB203580 (2 μ*M*). The inhibition of p38 MAPK signaling was shown to attenuate the HA-induced increase in the transcript levels for uPA (Figure 8B), cathepsin K (Figure 8C), and to abrogate the HA-induced expression of the *MT1-MMP *gene to below basal levels (Figure 8D).

## Discussion

Molecular stratification of gene-expression data has defined a minimum of five distinct subtypes of breast cancer [1]. Our analysis of breast tumor material determined that CD44 was inversely correlated with expression of the ER and PR, with no correlation to Her2, and that enrichment of CD44 was associated with BL-BCa, the subtype associated with the poorest clinical outcome. Our tissue-based characterization is consistent with other recent studies reporting the localization of CD44 in BL-BCa [6]. The association of CD44 with the most aggressive subtype of disease is consistent with the well-established cellular functions of CD44 in promoting cell invasion and cell adhesion *in vitro*, and the capacity for CD44 to increase spontaneous metastasis of breast cancer cells *in vivo *[7,8; McFarlane S, Coulter J, Waugh DJJ, unpublished observations]. Bourguignon and colleagues [30-32] provided a detailed understanding of CD44-promoted activation of Rho-GTPase family signaling, underpinning the reorganization of the cell cytoskeleton to facilitate the active migration of cells. The Stamenkovic and Seiki laboratories also reported that CD44 can complex with secreted and membrane-tethered matrix metalloproteinases, localizing their proteolytic activity to the invasive edge of tumor cells [11-14]. In this study, we now add a further dimension to our understanding of CD44-promoted invasion of breast cancer. In a series of experiments, conducted primarily in MDA-MB-231 breast cancer cells, but supported by additional observations in other well-established models of ER-negative and BL-BCa, we show that HA and CD44 can upregulate expression of key proteases that underpin the invasion of BL-BCa cells through experimental matrices. Importantly, our studies demonstrate that CD44 affects the expression and activity of members of the serine protease, cysteine cathepsin, and matrix metalloproteinase family. Collectively, these enzymes may contribute a spatial and temporal function throughout the metastatic cascade, providing invasive breast cancer cells with the essential complement of proteolytic activity to traverse successfully the physical barriers provided by the tissue matrix and basement membranes in both the primary and secondary tissue sites.

The serine protease uPA and its inhibitor PAI-1 are *bona fide *markers of poor prognosis that predict distant metastasis in breast cancer [15,16], whereas expression of uPA is upregulated in tumor-initiating breast cancer cells [33]. Highly invasive MDA-MB-231Hi cells were shown to have elevated expression of uPA, uPAR, and PAI-1 at transcript and protein levels, and had elevated cell surface-associated and extracellular uPA activity. We also detected increased plasmin activity in the MDA-MB-231Hi cells, consistent with a more-efficient uPA-mediated cleavage of its substrate plasminogen. The addition of HA further increased the mRNA transcript levels for uPA, uPAR, and PAI-1 and increased the levels of the corresponding proteins in a time-dependent manner in these BL-BCa cells. Coincident with the increased expression of uPA and uPAR, HA increased the cell surface-associated activity of uPA in these cells.

Conversely, the loss of CD44 in MDA-MB-231Hi cells coincided with a decreased expression of uPA, uPAR, PAI-1, and PAI-2, and attenuated the capacity of HA to increase expression of uPA. Consequently, CD44-depleted cells were associated with a decreased level of plasmin activity.

The importance of these serine proteases to BL-BCa cell invasion was investigated *in vitro*. Consistent with the HA-induced promotion of initially uPA and subsequently plasmin activity, the serine protease inhibitor aprotinin abrogated the HA-induced invasion of the MDA-MB-231Hi cells through Matrigel. Similarly, RNAi-mediated knockdown of uPA attenuated the invasion of these BL-BCa cells, whereas an inhibition of MDA-MB-231Hi cell invasion was also observed by using a small-molecule inhibitor of uPA. In addition to this role for uPA in promoting localized invasion of BL-BCa cells, increased uPA activity was detected in highly disseminating tumor cells and implicated in regulating the intravasation of these disseminating cells [23-25]. Accordingly, CD44-positive BL-BCa cells may be more efficient in completing intravasation, consistent with the role of CD44 in promoting the spontaneous metastasis of breast cancer *in vivo *[8] and the increased detection of CD44-enriched cells to disseminate to distant organs [9,10]. Targeting of the enzymatic activity of uPA by using small-molecule inhibitors may therefore be an appropriate and effective adjuvant therapeutic strategy to use in BL-BCa, or those breast cancers showing strong expression of CD44, with the intent to reduce distant metastasis and prolong survival in this poor-prognosis group of patients. Importantly, the small-molecule inhibitor used in this study was capable of antagonizing both cell-surface and extracellular activities of uPA, suggesting that its inhibitory potential is not restricted by specific localization of the enzyme.

Collagen is a major constituent of the extracellular matrix of primary and secondary tumor sites. MDA-MB-231Hi cells showed increased capacity to degrade and invade through collagen, a response also attenuated by CD44 knockdown. As uPA has no capacity to degrade collagen directly, a further analysis characterized additional differential protease expression consistent with this increased collagen-degrading activity in MDA-MB-231Hi cells. Immunoblotting confirmed increased expression of two potent collagen-targeting proteases, MT1-MMP and cathepsin K, in the more invasive cells. Furthermore, HA increased (a) the transcript levels of each of the MT1-MMP and cathepsin K genes, (b) increased expression of the corresponding proteins, and (c) promoted the extracellular secretion of cathepsin K. As before, depletion of CD44 reduced the expression of MT1-MMP and the secretion of cathepsin K from the MDA-MB-231Hi cells, consistent with an overall reduction in the capacity of these cells to degrade collagen in the absence of CD44. Furthermore, loss of CD44 attenuated the HA-induced increases in protease expression, underlying the importance of this receptor in mediating the response. Loss of either MT1-MMP or cathepsin K enzymatic activity was shown to reduce the HA-promoted invasion of MDA-MB-231Hi cells through collagen.

MT1-MMP promotes the cleavage of the ectodomain of CD44 [13,14]. Our data suggest the existence of a positive-feedback mechanism by which the engagement of extracellular HA fragments with CD44 induces the transcription of the protease responsible for promoting the shedding of CD44 from the cell surface. Overall, CD44 cleavage would attenuate cell attachment to HA in the matrix and facilitate cell invasion. Moreover, CD44, MT1-MMP, and cortactin have been shown to localize to invadopodia [34], the membrane protrusions that localize enzymes required for ECM degradation at the leading edge of invasive cells. We suggest that CD44 may play an important hierarchic role in facilitating invadopodia organization; for example, we showed that CD44/HA-signaling increases the transcription and expression of both MT1-MMP and *EMS1*/cortactin in invasive BL-BCa cells [7]. Consistent with the past observations of Bourguignon and colleagues [30], we also confirmed that HA-induced CD44 signaling induces a posttranslational activation of cortactin signaling in these invasive breast cancer cells [30, McFarlane *et al*., unpublished data]. The capacity for CD44 to increase MT1-MMP expression and promote its localization within invadopodia would be consistent with the capacity of CD44 to induce breast cancer cell invasion. MT1-MMP has multiple substrates within the extracellular matrix, including collagen I, II, and III; fibronectin; and each of laminin 1 and 5; in addition to having an enzyme convertase function that leads to an activation of the gelatinase MMP-2 and the collagenase MMP-13 [26,35]. Consequently, MT1-MMP activity is associated with localized invasion but also contributes to the degradation of basement membranes, a process essential to successful intravasation and extravasation of tumor cells. The capacity for CD44 to increase an enzyme that can activate a downstream cascade of further MMP activity would also be consistent with the role of this receptor in enabling cells to complete metastasis to different tissue sites.

Cathepsin K is a member of the papain/cysteine protease superfamily [27]. Cathepsin K expression has previously been reported in primary human breast tumors and their metastases, including breast cancer cells within bone metastatic lesions [36]. Analysis of bone metastases has reported CD44 expression on resident breast cancer cells [9], whereas recent data from our laboratory confirm that loss of CD44 on systemically administered MDA-MB-231 cells reduces secondary tumor formation in athymic nude mice, including a reduction in osteolytic metastases [McFarlane *et al*., unpublished data]. Furthermore, the inhibition of hyaluronan synthesis in MDA-MB-231 cells has been reported to reduce subsequent bone metastasis *in vivo *[37]. Thus CD44-promoted increases in cathepsin K expression and secretion may assist the initial invasion and colonization of BrCa cells within collagen-enriched secondary sites, including the bone.

In summary, we define the importance of CD44 in underpinning the invasion of BL-BCa cells, and illustrate the importance of HA and CD44 in upregulating a diverse spectrum of protease expression and activity that can function in a spatial or temporal manner to enable invasive cells to remodel their localized environment or perform specialist functions, including the successful completion of intravasation and extravasation. The capacity of CD44 to induce protease activity is consistent with the aggressive clinical characteristics of BL-BCa and the increased propensity to invade locally through primary breast tissue and to colonize collagen-enriched organs, including the liver, lungs, brain, and skin. Thus the detection of high CD44 expression in tumor biopsy tissue may be a suitable biomarker to identify patients who may benefit from the provision of MT1-MMP- or uPA-targeting therapeutics to reduce the risk of intravasation and distant metastasis. Moreover, as CD44-enriched "stem cell-like" breast cancer cells have been shown to disseminate early in the course of disease [9,10], inhibitors of these key protease activities may be a relevant adjuvant approach to counteract the successful invasion and colonization of secondary tissues by these invasive breast cancer cells.

## Abbreviations

BL-BCa: basal-like breast cancer; HA: hyaluronan; MMP: matrix metalloproteinase; PAI-1/2: plasminogen activator inhibitor-1/2; RNAi: small interfering RNA; shRNA: short-hairpin RNA; TMA: tissue microarray; uPA: urokinase plasminogen activator; uPAR: urokinase plasminogen activator receptor.

## Competing interests

DJJW is a consultant and member of the Scientific Advisory Board for Almac Discovery, who have developed a peptide-based inhibitor of angiogenesis whose mechanism is mediated in a CD44-depedent manner (Valentine *et al*., *Clin Cancer Res *2011). The results presented in this article are not infringed by this interest, and no funding was provided by Almac Discovery to undertake this research. None of the additional authors of the article declare any competing interests.

## Authors' contributions

NM, AH, and JN conducted the majority of *in vitro *experimentation and data analysis, with a minor contribution from SMcF. SMcF, AO'G, and SC undertook the tissue analysis, which was assisted by an independent pathological review conducted by KJ and EWK. DJJW conceived of the study hypothesis, assisted with experimental design and data interpretation, and wrote the manuscript with editorial assistance from AH and SMcF. All authors read and approved the manuscript for publication.

## Supplementary Material

Additional file 1 **Validation of the siRNA-mediated knockdown of CD44 and MT1-MMP**. **(A) **Representative immunoblot showing the reduction in CD44 expression in MDA-MB-231Hi cells, by using increasing concentrations of a CD44-targeting RNAi-oligonucleotide or a nontargeting (NT) RNA-oligonucleotide. **(B) **Representative immunoblot showing MT1-MMP protein levels detected in MDA-MB-231Hi cells transfected with RNAi-oligonucleotides targeting these proteins. Cells were also treated with an NT RNAi oligonucleotide as a control. Equal protein loading in immunoblots was confirmed by reprobing the membranes for β-tubulin. Click here for file

Additional file 2 **Characterization of HA-induced gene transcription in Hs578T cells**. Bar graph illustrating the relative change in mRNA transcript levels in uPA, uPAR, and PAI-1 in the Hs578T BL-BCa cell line, in response to stimulation with 100 μg/ml HA. Data shown are the mean ± SEM value calculated from three independent experiments. Statistically significant differences between data points in quantitative assays were determined by using a Student two-tailed *t *test. *P < 0.05; **P < 0.01; ***P < 0.001. Click here for file

Additional file 3 **Characterization of a CD44-depleted clone of the highly invasive breast cancer cells and the relation to uPA signaling components**. **(A) **Bar graph showing decreased CD44 mRNA transcript levels present in the MDA-MB-231 sh#1 cells relative to MDA-MB-231 NT cells (*P *< 0.001; *n *= 4). **(B) **Representative immunoblot showing knockdown of CD44s expression in the MDA-MB-231 sh#1 cells compared with the MDA-MB-231 NT cell line. The blots were reprobed with β-tubulin as a loading control. **(C) **Bar graph illustrating the attenuated invasive potential of MDA-MB-231 sh#1 cells through Matrigel relative to the MDA-MB-231 NT cells (*P *< 0.05; *n *= 3). **(D) **Bar graphs showing decreased mRNA transcript levels of uPA, uPAR, PAI-1, and PAI-2 in the MDA-MB-231 sh#1 cells relative to MDA-MB-231 NT cells (*P *< 0.001 for all genes; data shown are from a minimum of four independent experiments). **(E) **Immunoblots demonstrating the decreased expression of uPA, uPAR, PAI-1, and PAI-2 protein in MDA-MB-231 cells in which CD44 expression had been decreased by using a short-hairpin strategy (MDA-MB-231 sh#1) relative to nontargeting control cells (MDA-MB-231 NT). All data points shown are mean ± SEM, and statistically significant points were determined by using a Student two-tailed *t *test. *P < 0.05; ***P < 0.001. Click here for file

Additional file 4 **Characterization of the siRNA-mediated knockdown of uPA in highly invasive breast cancer cells**. **(A) **uPA RNAi validation in the MDA-MB-231Hi cell line. Immunoblot shows a substantial reduction in uPA protein levels present in MDA-MB-231Hi cells transfected with 200, 150, and 75 n*M *concentrations of uPA RNAi. Cells were also treated with an NT RNAi oligonucleotide at each concentration as a control. Blots were reprobed with GAPDH as a loading control. **(B) **Bar graph showing a significant reduction in uPA mRNA levels present in MDA-MB-231Hi cells after transfection with 200 n*M *uPA RNAi relative to NT RNAi-treated cells (*P *< 0.001; *n *= 4). Blots were reprobed with β-tubulin as a loading control. Statistically significant points were determined by using a Student two-tailed *t *test. ***P < 0.001. Click here for file

Additional file 5 **CD44 signaling potentiates cathepsin K expression in the MDA-MB-157 BL-BCa cell line**. q-PCR was conducted on RNA isolates extracted from MDA-MB-157 cells over a 12-hour time course after stimulation with HA (100 μg/ml). Data shown represent HA-mediated increases in the mRNA expression of cathepsin K. Data points are the mean ± SEM fold-change relative to control, determined from three independent experiments. Click here for file
