# Supplementary figures and images for: CD44 enhances invasion of basal-like breast cancer cells by upregulating serine protease and collagen-degrading enzymatic expression and activity
Source: Breast Cancer Res. 2012 May 23;14(3):R84. doi: 10.1186/bcr3199 (PMC3446347; doi:10.1186/bcr3199)

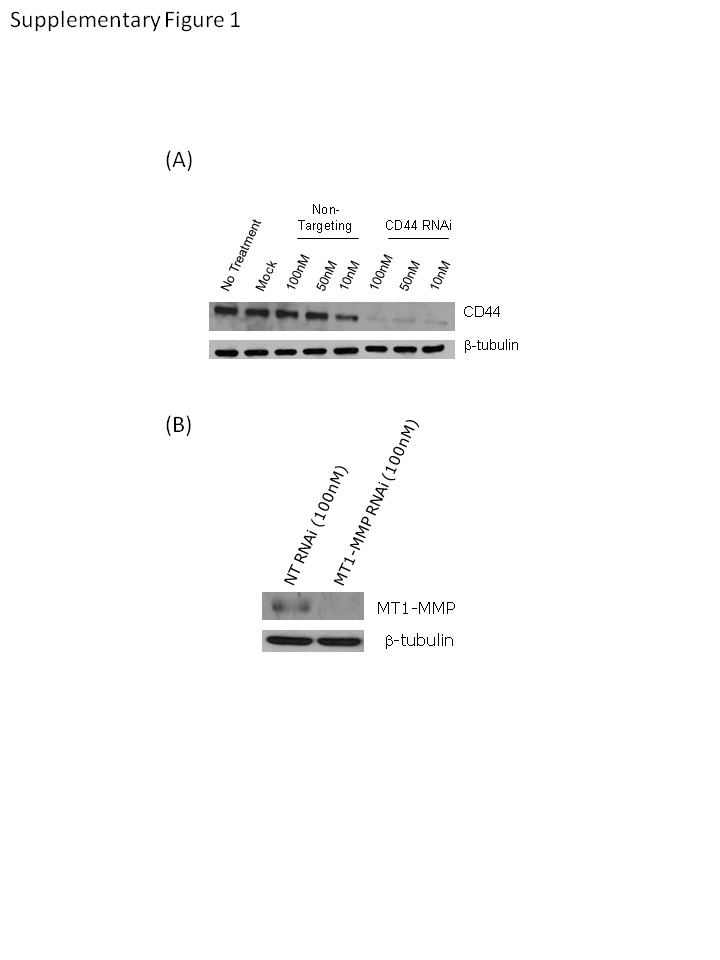

Supplement: Additional file 1 — Validation of the siRNA-mediated knockdown of CD44 and MT1-MMP. (A) Representative immunoblot showing the reduction in CD44 expression in MDA-MB-231Hi cells, by using increasing concentrations of a CD44-targeting RNAi-oligonucleotide or a nontargeting (NT) RNA-oligonucleotide. (B) Representative immunoblot showing MT1-MMP protein levels detected in MDA-MB-231Hi cells transfected with RNAi-oligonucleotides targeting these proteins. Cells were also treated with an NT RNAi oligonucleotide as a control. Equal protein loading in immunoblots was confirmed by reprobing the membranes for β-tubulin. [file bcr3199-S1.TIFF]

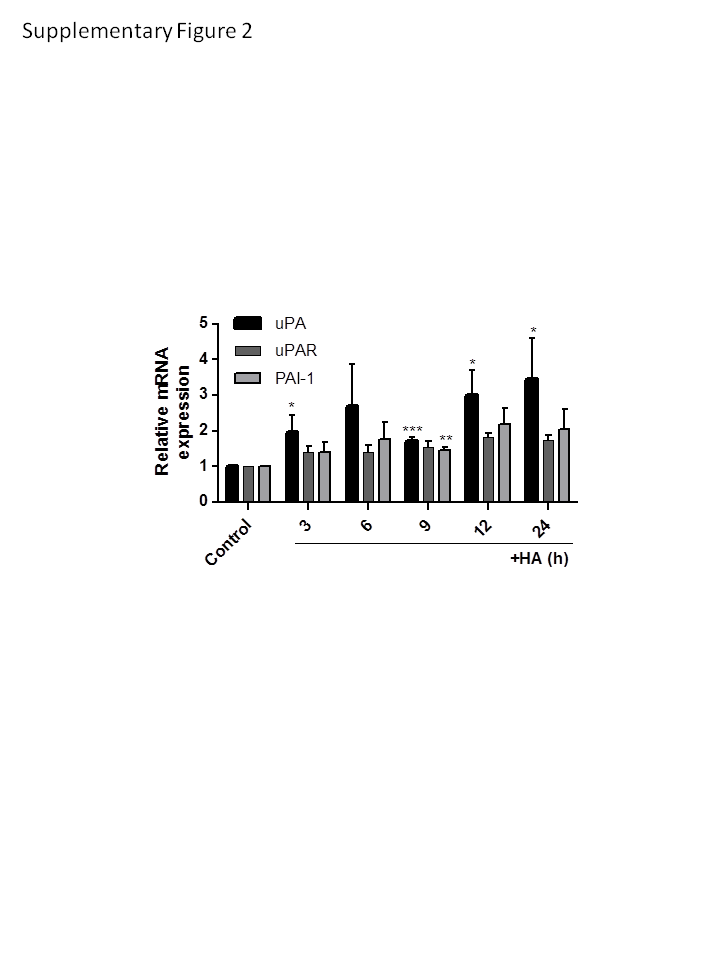

Supplement: Additional file 2 — Characterization of HA-induced gene transcription in Hs578T cells. Bar graph illustrating the relative change in mRNA transcript levels in uPA, uPAR, and PAI-1 in the Hs578T BL-BCa cell line, in response to stimulation with 100 μg/ml HA. Data shown are the mean ± SEM value calculated from three independent experiments. Statistically significant differences between data points in quantitative assays were determined by using a Student two-tailed t test. *P < 0.05; **P < 0.01; ***P < 0.001. [file bcr3199-S2.TIFF]

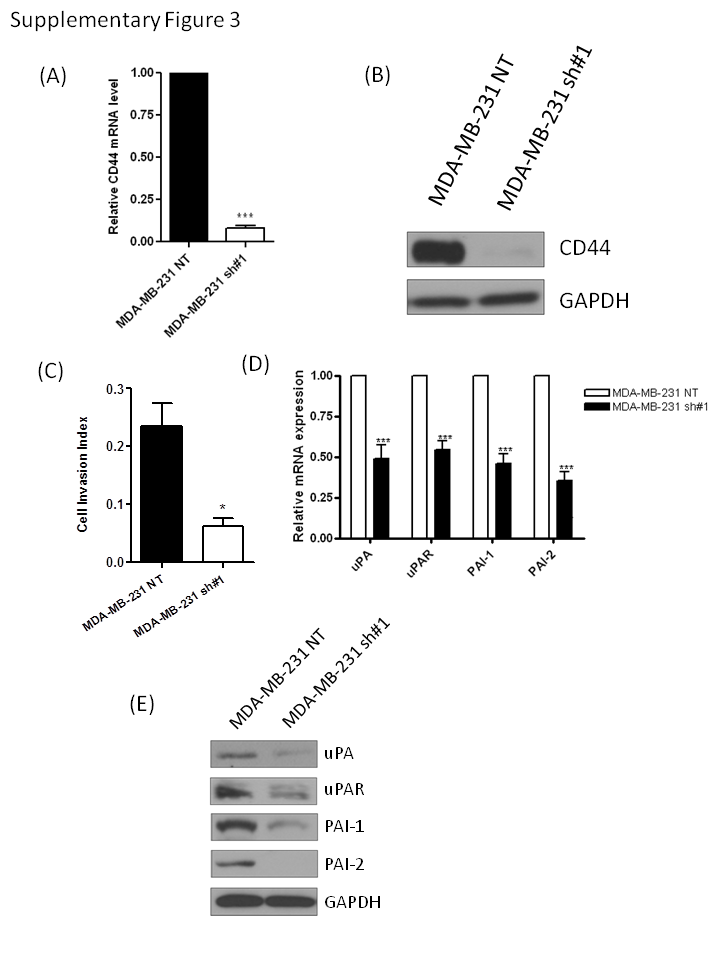

Supplement: Additional file 3 — Characterization of a CD44-depleted clone of the highly invasive breast cancer cells and the relation to uPA signaling components. (A) Bar graph showing decreased CD44 mRNA transcript levels present in the MDA-MB-231 sh#1 cells relative to MDA-MB-231 NT cells (P < 0.001; n = 4). (B) Representative immunoblot showing knockdown of CD44s expression in the MDA-MB-231 sh#1 cells compared with the MDA-MB-231 NT cell line. The blots were reprobed with β-tubulin as a loading control. (C) Bar graph illustrating the attenuated invasive potential of MDA-MB-231 sh#1 cells through Matrigel relative to the MDA-MB-231 NT cells (P < 0.05; n = 3). (D) Bar graphs showing decreased mRNA transcript levels of uPA, uPAR, PAI-1, and PAI-2 in the MDA-MB-231 sh#1 cells relative to MDA-MB-231 NT cells (P < 0.001 for all genes; data shown are from a minimum of four independent experiments). (E) Immunoblots demonstrating the decreased expression of uPA, uPAR, PAI-1, and PAI-2 protein in MDA-MB-231 cells in which CD44 expression had been decreased by using a short-hairpin strategy (MDA-MB-231 sh#1) relative to nontargeting control cells (MDA-MB-231 NT). All data points shown are mean ± SEM, and statistically significant points were determined by using a Student two-tailed t test. *P < 0.05; ***P < 0.001. [file bcr3199-S3.TIFF]

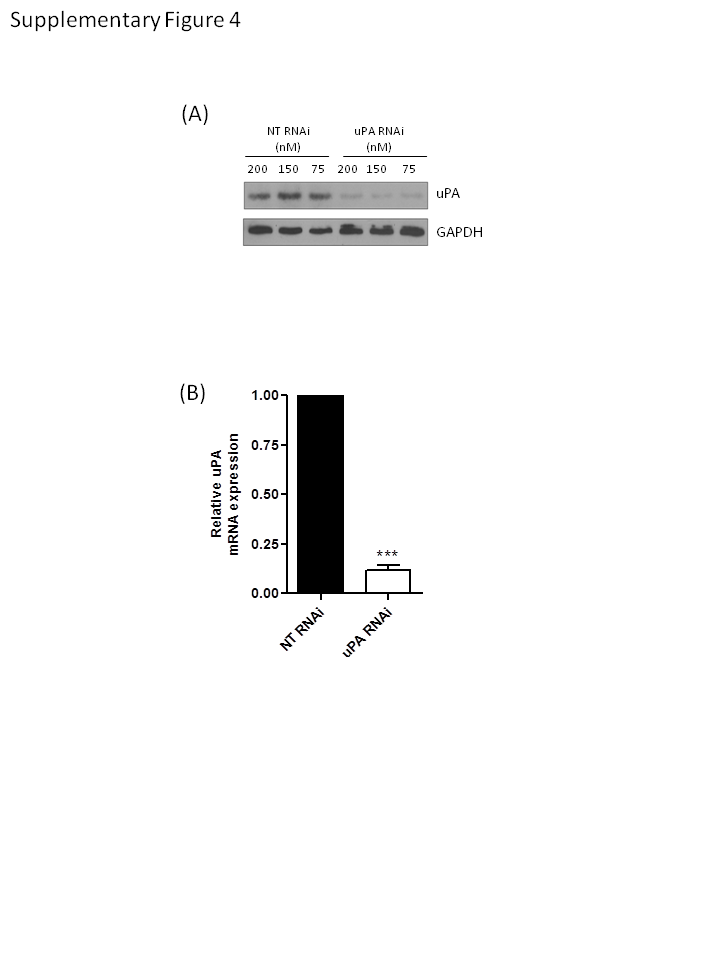

Supplement: Additional file 4 — Characterization of the siRNA-mediated knockdown of uPA in highly invasive breast cancer cells. (A) uPA RNAi validation in the MDA-MB-231Hi cell line. Immunoblot shows a substantial reduction in uPA protein levels present in MDA-MB-231Hi cells transfected with 200, 150, and 75 nM concentrations of uPA RNAi. Cells were also treated with an NT RNAi oligonucleotide at each concentration as a control. Blots were reprobed with GAPDH as a loading control. (B) Bar graph showing a significant reduction in uPA mRNA levels present in MDA-MB-231Hi cells after transfection with 200 nM uPA RNAi relative to NT RNAi-treated cells (P < 0.001; n = 4). Blots were reprobed with β-tubulin as a loading control. Statistically significant points were determined by using a Student two-tailed t test. ***P < 0.001. [file bcr3199-S4.TIFF]

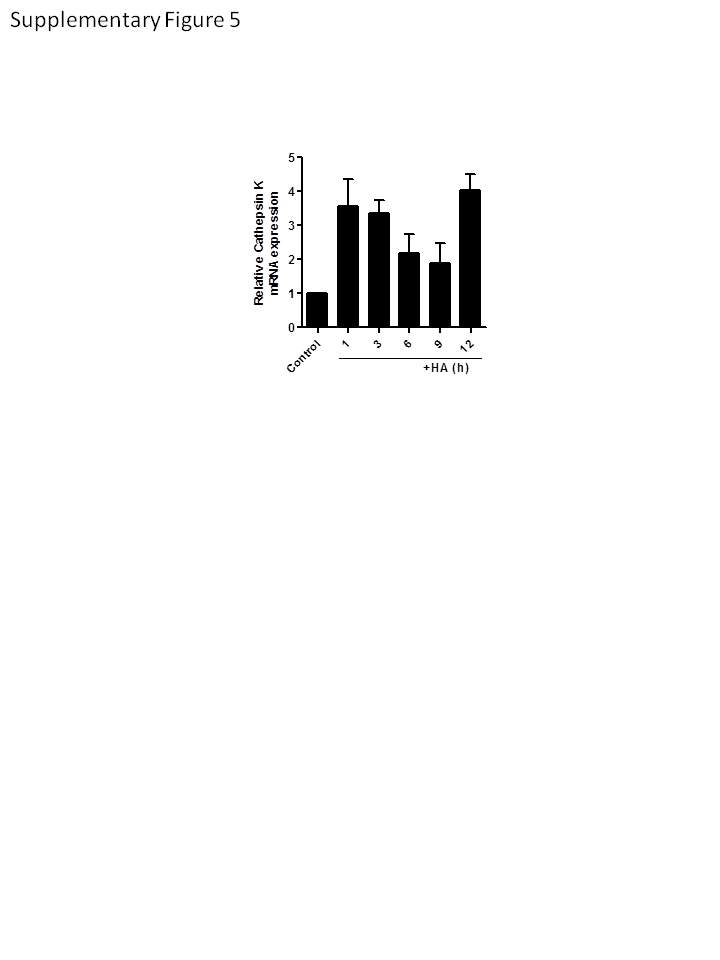

Supplement: Additional file 5 — CD44 signaling potentiates cathepsin K expression in the MDA-MB-157 BL-BCa cell line. q-PCR was conducted on RNA isolates extracted from MDA-MB-157 cells over a 12-hour time course after stimulation with HA (100 μg/ml). Data shown represent HA-mediated increases in the mRNA expression of cathepsin K. Data points are the mean ± SEM fold-change relative to control, determined from three independent experiments. [file bcr3199-S5.TIFF]
